# High-Resolution Genome-Wide Dissection of the Two Rules of Speciation in *Drosophila*

**DOI:** 10.1371/journal.pbio.0050243

**Published:** 2007-09-11

**Authors:** John P Masly, Daven C Presgraves

**Affiliations:** Department of Biology, University of Rochester, Rochester, New York, United States of America; University of Edinburgh, United Kingdom

## Abstract

Postzygotic reproductive isolation is characterized by two striking empirical patterns. The first is Haldane's rule—the preferential inviability or sterility of species hybrids of the heterogametic (XY) sex. The second is the so-called large X effect—substitution of one species's X chromosome for another's has a disproportionately large effect on hybrid fitness compared to similar substitution of an autosome. Although the first rule has been well-established, the second rule remains controversial. Here, we dissect the genetic causes of these two rules using a genome-wide introgression analysis of Drosophila mauritiana chromosome segments in an otherwise D. sechellia genetic background. We find that recessive hybrid incompatibilities outnumber dominant ones and that hybrid male steriles outnumber all other types of incompatibility, consistent with the dominance and faster-male theories of Haldane's rule, respectively. We also find that, although X-linked and autosomal introgressions are of similar size, most X-linked introgressions cause hybrid male sterility (60%) whereas few autosomal introgressions do (18%). Our results thus confirm the large X effect and identify its proximate cause: incompatibilities causing hybrid male sterility have a higher density on the X chromosome than on the autosomes. We evaluate several hypotheses for the evolutionary cause of this excess of X-linked hybrid male sterility.

## Author Summary

The evolution of reproductive isolation is a fundamental step in the origin of species. One kind of reproductive isolation, the sterility and inviability of species hybrids, is characterized by two of the strongest rules in evolutionary biology. The first is Haldane's rule: for species crosses in which just one hybrid sex is sterile or inviable, it tends to be the sex defined by having a pair of dissimilar sex chromosomes (e.g., the “XY” of males in humans). The second rule is the large X effect: the X chromosome has a disproportionately large effect on hybrid fitness. We dissected the genetic causes of these two rules of speciation by replacing many small chromosomal segments of the fruit fly Drosophila sechellia with those of a closely related species, D. mauritiana. Together, these segments cover 70% of the genome. We found that virtually all segments causing hybrid sterility or inviability act recessively and that hybrid male sterility is by far the most common type of hybrid incompatibility, confirming two leading theories about the causes of Haldane's rule. We also found that X-linked segments are more likely to cause hybrid male sterility than similarly sized autosomal segments. These results show that the large X effect is caused by a higher density of hybrid incompatibilities on the X chromosome.

## Abstract 3

A genome-wide introgression analysis of *Drosophila mauritiana* chromosome segments in an otherwise *D. sechellia* genetic background confirms the large X effect, a cornerstone of speciation theory, and reveals its cause.

## Introduction

Speciation occurs when two populations become reproductively isolated from each other through the evolution of one or more barriers to gene flow [1,2]. One of the most intensively studied forms of reproductive isolation is intrinsic postzygotic isolation, the inviability or sterility of species hybrids. A model describing the evolution of hybrid inviability and hybrid sterility was proposed independently by Dobzhansky [1] and Muller [3]. The essence of their idea is that divergence at interacting loci between species can cause deleterious, incompatible epistatic interactions in interspecific hybrids. Genetic studies have now amassed abundant evidence that these hybrid incompatibilities are a common cause of intrinsic hybrid fitness problems [4].

In 1989, Coyne and Orr suggested that “two rules of speciation” further characterize the genetics of postzygotic isolation [5]. The first is Haldane's rule, which states that when hybrids of just one sex are dead or sterile, it is usually the heterogametic (XY) sex [6]. This rule is widely obeyed in both male-heterogametic (XY; e.g., *Drosophila* and mammals) and female-heterogametic (ZW; e.g., *Lepidoptera* and birds) taxa [7–12]. After two decades of intensive study, most speciation geneticists now agree that Haldane's rule is caused by a combination of at least two phenomena [4].

First, the dominance theory posits that the alleles causing hybrid incompatibilities are, on average, partially recessive for their effects on hybrid fitness [3,13–16]. Thus, heterogametic F_1_ hybrids (hereafter XY males) experience the full effects of all recessive X-linked hybrid incompatibilities, whereas homogametic F_1_ hybrids experience few or none. The main prediction of the dominance theory is supported by both genetic [17–27] and comparative studies [28,29].

Second, the faster-male theory posits that incompatibilities causing hybrid male sterility accumulate faster than those causing hybrid inviability or hybrid female sterility [8,30]. Two processes might give rise to such faster-male evolution. First, sexual selection on male-specific genes could increase divergence at these loci, increasing the chance of hybrid male sterility. Second, spermatogenesis itself might be an inherently sensitive developmental process that is easily perturbed in hybrids. Regardless of its underlying causes, evidence of faster-male evolution has been obtained from genetic studies [21,22,26,31], hybrid gene misexpression studies [32–34], and comparative analyses [29].

Two other phenomena, faster evolution of X-linked loci (the faster-X theory [35]; but see [36,37]) and some forms of genetic conflict [31,38–42], have also been suggested as causes of Haldane's rule, but their general importance remains unclear.

The second rule of speciation is the so-called large X effect [5,7,35]. In backcross analyses of species hybrids, substitution of one species's X chromosome for the other's has a disproportionately large effect on hybrid fitness relative to similar substitution of an autosome. The large X effect (recently dubbed “Coyne's rule” [43]) has been observed in genetic analyses of hybrid inviability [44–46] and hybrid sterility [47–58], and also has been inferred from patterns of gene flow across natural hybrid zones: X-linked loci often pass across hybrid zone boundaries less freely than do autosomal loci [59–64]. Despite these observations, the causes, and indeed the existence, of the large X effect remain controversial. Wu and Davis [8] pointed out that backcross analyses of hybrid males compare the hemizygous effects of X chromosomes with the heterozygous effects of autosomes: hybrid males suffer from all recessive incompatibilities on the X while those on the autosomes remain mostly masked. They argued that the large X effect, although expected under the dominance theory [10,16], arises as a consequence of the backcross design and signifies nothing special about the X chromosome per se. However, the large X effect could also result from a higher density of hybrid incompatibility loci on the X [5,8,9,35]. Therefore, to distinguish the relative contributions of dominance versus density to the large X effect, hemizygous X chromosome segments must be compared to homozygous autosomal segments [8].

Several studies have performed this test with mixed results. Hollocher and Wu [22] introgressed three regions from both the D. sechellia and the D. mauritiana second chromosome into a largely D. simulans genetic background and compared the homozygous effects of the autosomal introgressions on postzygotic reproductive isolation to those of hemizygous X-linked introgressions. Their results showed that homozygous autosomal introgressions do, in fact, have effects on hybrid inviability and hybrid sterility similar in magnitude to X-linked introgressions. They concluded that, to their level of resolution, the large X effect is indeed a methodological consequence of dominance. Two other studies in *Drosophila* have, however, found tentative evidence for a higher density of hybrid male sterility on the X. True et al. [21] performed a genome-wide screen for hybrid incompatibilities between D. mauritiana and D. simulans. Using a collection of D. mauritiana lines bearing selectable *P*-element markers at 87 known cytological positions, they generated 355 homozygous introgressions after backcrossing to D. simulans for 15 generations. Their results showed that D. mauritiana introgressions on the X cause hybrid male sterility 50% more often than those on the autosomes. More recently, Tao et al. [26] used a fine-scale mapping approach in the same hybridization to study the distribution of hybrid incompatibilities by generating 218 overlapping D. mauritiana introgressions covering the third chromosome. Although they did not create a similar set of D. mauritiana introgressions on the X, Tao et al. compared the effects of their third chromosome introgressions with previously published data from 265 X-linked introgressions used to map hybrid male sterility between these two species. These comparisons suggested that the X carries ∼2.5 times more hybrid sterility loci than the autosomes. Thus, both True et al. and Tao et al. tentatively concluded that the X chromosome has a higher density of hybrid male sterility factors.

There are two possible explanations for the conflicting results between these three studies. The first involves introgression size. If larger introgressions are more likely to cause hybrid incompatibilities [65], then large introgressions would upwardly bias estimates of the number of hybrid incompatibilities regardless of their location. For example, Hollocher and Wu compared autosomal introgressions roughly the size of a chromosome arm (∼20–30 Mb) with X-linked introgressions that were as small as one-third of the size of their autosomal introgressions (and collected from a different experiment). This difference in introgression size might have led them to overestimate the relative effects of the second chromosome introgressions. Although True et al.'s data suggested that no obvious difference in size existed between their X-linked and autosomal introgressions, the authors urged caution in the interpretation of their results, as they were unable to systematically obtain introgression size estimates. The second possible explanation involves sampling bias. Tao et al. used X-linked introgressions in their comparison that were significantly smaller than their third chromosome introgressions. Although this appears to argue in favor of the large X effect, these X-linked introgressions were not a random sample: they came from regions known from previously published work to have large effects on hybrid male sterility (see [66–69]). Thus, to date, there has not been an analysis that simultaneously phenotypes and genotypes many X-linked and autosomal introgressions from the same experiment.

The large X effect hypothesis makes a clear prediction: introgressions of a given size will be incompatible more often when they reside on the X chromosome than when they reside on an autosome. Because introgression size affects hybrid fitness, it is important to compare introgressions of modest size on the X versus the autosomes. Here, we take an approach similar to that used by True et al., but we study the genome-wide distribution of hybrid incompatibilities between D. mauritiana and *D. sechellia,* two island endemic species of fruit flies that diverged ∼300,000 y ago [70]. Using a collection of *D. mauritiana P*-element insertion lines, we generated 142 independent introgressions that collectively cover ∼70% of the genome. We use these data to test theories about the genetic causes of the two rules of speciation: Haldane's rule and the large X effect.

## Results/Discussion

### Generating the *D. mauritiana–D. sechellia* Introgression Lines

**Figure 1:**
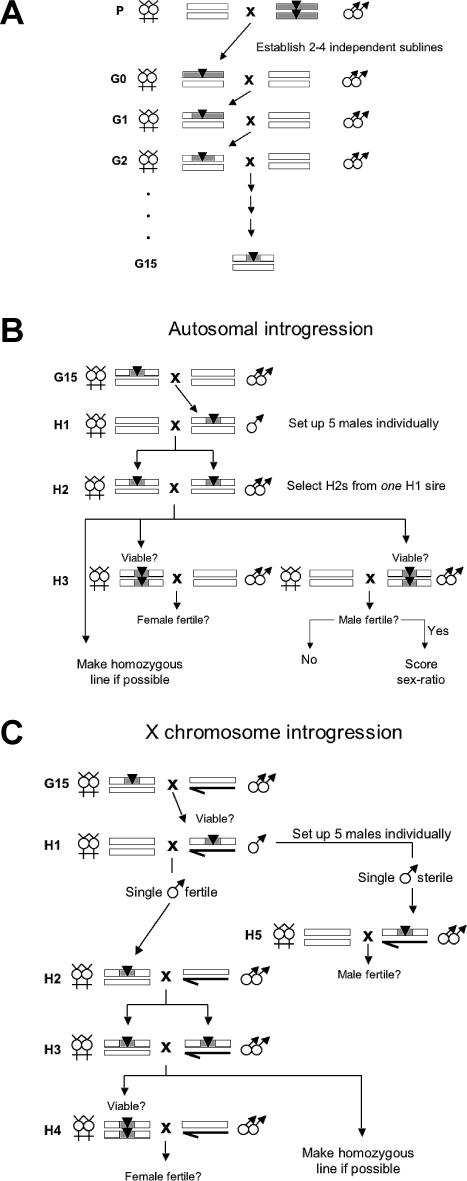
Introgression Construction *D. mauritiana P*[*w* ^+^]-marked chromosome segments were introgressed into a *D. sechellia w* background via 15 generations of backcrossing (A). The *P*[*w* ^+^] insertion carries a *mini-white* gene that acts as a partially dominant visible eye-color marker for selecting heterozygous females for each backcross. Introgressions were made homozygous on the autosomes (B) and the X chromosome (C), and tested for postzygotic hybrid incompatibilities.

Crosses between D. mauritiana and D. sechellia produce fertile F_1_ hybrid females and sterile F_1_ hybrid males in both directions of the cross. To place our D. mauritiana introgressions in an otherwise D. sechellia genetic and cytoplasmic background, all introgressions were initiated by crossing *D. sechellia white (w)* females with *D. mauritiana P*[*w* ^+^] males. Each *D. mauritiana P*[*w* ^+^] insertion line carries a *mini-white* gene, which acts as a semi-dominant visible eye-color marker, allowing us to select heterozygous *P*[*w* ^+^] females in a *D. sechellia w* background for each backcross. Following the introgression scheme shown in Figure 1 (the same crossing scheme used in [21]), we introgressed 66 different *P*[*w* ^+^]-marked chromosomal regions from D. mauritiana into a *D. sechellia w* genetic background via 15 generations of repeated backcrossing. On average we constructed 2.2 independent replicates per *P*[*w* ^+^] insert, for a total of 142 sublines. From each subline, we scored the viability and fertility of homozygous (hemizygous) introgressions (see Materials and Methods). An average of 156 flies were scored per subline, for a total of 22,128 flies. Of the 142 sublines, 55 (39%) show some form of postzygotic reproductive isolation (Table S1).

### The Genetic Basis of Haldane's Rule

Haldane's rule in male-heterogametic taxa is thought to result from the general recessivity of hybrid incompatibility alleles (the dominance theory) and the more rapid accumulation of incompatibilities that cause hybrid male sterility (the faster-male theory). Our introgression data support both theories. The crossing scheme used to create the introgressions required backcrossing through hybrid females that were heterozygous for D. mauritiana genetic material. Although a few sublines were lost during backcrossing to *D. sechellia w,* at least one subline per *P*[*w* ^+^] insert survived the introgression procedure. This suggests that there are few (if any) incompatibilities sufficiently dominant to cause strong sterility or inviability in heterozygous introgression females. Similarly, after 15 generations of backcrossing, hybrid males also appear unaffected by dominantly acting incompatibilities: all autosomal sublines produced viable heterozygous *P*[*w* ^+^] males (H1 males; see Figure 1B for notation), and 94% (526 of 562 H1 males tested) of these were fertile. Indeed, every autosomal subline produced at least two fertile H1 males of five tested. Thus, virtually all of the hybrid incompatibilities we detect act fairly recessively, consistent with the dominance theory.

**Figure 2:**
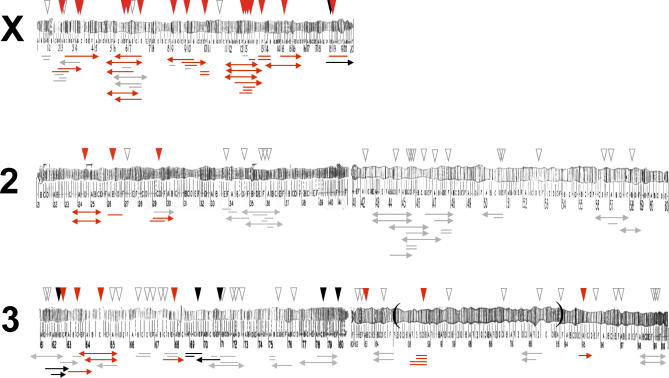
Distribution of D. mauritiana Introgressions in the D. sechellia Genome Inverted triangles above each chromosome show *P*[*w* ^+^] insertion sites: black indicates hybrid inviable; red indicates hybrid male-sterile; white indicates hybrid fertile or untested. Horizontal lines below each chromosome show the approximate sizes of 108 of 142 introgressions. Arrows indicate that D. mauritiana material extends beyond the marker resolution at that location. Black indicates hybrid inviable introgressions; red indicates hybrid male-sterile introgressions; gray indicates hybrid fertile introgressions. The breakpoints of an inversion difference on chromosome arm 3R between D. melanogaster and the D. simulans clade species *(D. simulans, D. mauritiana,* and *D. sechellia)* are shown as brackets.

Second, we find a dramatic excess of hybrid male sterility over hybrid inviability or hybrid female sterility (Figure 2). Of 142 sublines, only 9 (6%) cause hybrid inviability. The fertility of most introgressions could therefore be tested for both sexes in 133 sublines. Surprisingly, all viable sublines produce fertile hybrid females. This result differs from that of True et al. [21] who found that ∼6% of their *D. mauritiana–D. simulans* sublines were hybrid female-sterile. Most striking, however, is the large number of sublines that cause hybrid male sterility: 44 of 133 viable sublines (33%) produce completely sterile hybrid males. This excess of hybrid male-sterile introgressions over hybrid inviable or hybrid female-sterile introgressions supports the faster-male theory of Haldane's rule, and is consistent with other genetic studies in *Drosophila* [4,21,22,26].

The incompatibilities we observe could, however, be artifacts in two ways. First, deleterious mutations might be segregating within each parental stock. However, we observe inviability or sterility only in some introgression lines—not in the parental lines—which indicates that the incompatibilities detected result from incompatible interactions between genes from D. mauritiana and D. sechellia. Second, some of the inviability and sterility we observe might be caused by spontaneous mutations that arose during the introgression procedure. Previous estimates show that mutations account for 1%–5% of lethal sublines and ∼0.2%–2.5% of sterile sublines seen in *D. mauritiana–D. simulans* hybrids [21,26]. Although our study was performed in *D. mauritiana–D. sechellia* hybrids, there is no evidence of a difference in mutation rates between hybrids of these three species [71]. Spontaneous mutation cannot, therefore, account for the relative abundances of the different classes of hybrid incompatibilities.

Two additional facts militate against spontaneous mutation: (1) the rate of spontaneous mutation to lethality is several-fold higher than that to male sterility in *Drosophila* [72,73] and thus cannot account for the excess of hybrid male-sterile introgressions compared to hybrid inviable or hybrid female-sterile introgressions, and (2) hybrid incompatibilities were typically confirmed with replicate sublines (20 of 32 *P*[*w* ^+^] inserts). Even if we exclude singly incompatible sublines from our data, our results remain qualitatively the same: a large proportion of D. mauritiana introgressions cause hybrid male sterility in a D. sechellia genetic background.

### The Genetic Basis of the Large X Effect for Hybrid Male Sterility

Introgressions causing hybrid male sterility are not randomly distributed throughout the genome (Figure 2). Instead, we find that the X chromosome possesses a significant excess of hybrid male steriles compared to the autosomes: 60% (27 of 45) of X-linked introgressions are hybrid male-sterile, whereas only 18% (17 of 97) of autosomal introgressions are hybrid male-sterile (*χ* ^2^ = 25.9, *p* < 0.0001). Although this pattern is consistent with that predicted by the large X effect, it could have several trivial causes. Two of these can be ruled out. First, we can exclude a clustering of *P*[*w* ^+^] inserts in male-sterile regions on the X as the cause of this pattern, as the collection of *P*[*w* ^+^] inserts was shown previously to have a random distribution within D. mauritiana chromosome arms [21,74]. Second, we can exclude the possibility that *P*[*w* ^+^] inserts in hybrid male-sterile regions on the X are represented by more sublines than those in fertile regions (which would inflate the fraction of sterile X-linked introgressions), as the number of sublines scored for sterile and fertile *P*[*w* ^+^] inserts is the same on both the X (*χ* ^2^ = 0.01, *p =* 0.91) and the autosomes (*χ* ^2^ = 0.18, *p =* 0.67).

**Figure 3:**
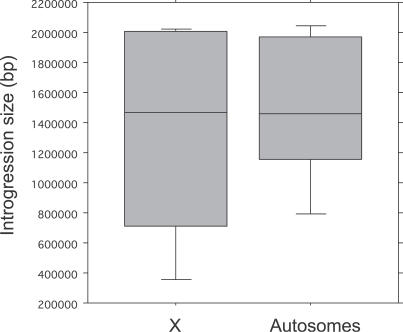
Distribution of X Chromosomal and Autosomal Introgression Sizes Boxes show the interquartile range; vertical lines are drawn out to the extreme values; horizontal lines within boxes show the median.

Last, the apparent excess of hybrid male-sterile sublines on the X chromosome could result from systematically larger introgressions on the X versus the autosomes. To test this possibility, we estimated the size of our introgressions by genotyping three microsatellite markers on each side of the *P*[*w* ^+^] insert in 108 sublines from 55 *P*[*w* ^+^] inserts. The three markers on each side were spaced ∼50 kb, ∼500 kb, and ∼1 Mb away from the *P*[*w* ^+^] insert (see Materials and Methods). For 67 of these sublines, we could reliably score or infer the genotype at all six markers, hereafter referred to as “complete” sublines. For the remaining 41 sublines, we were able to obtain only partial genotypes because of repeated PCR failure or a lack of diagnostic markers. Our results show that X-linked and autosomal introgressions appear similar in size (Figure 3).

We nevertheless tested for potential size differences in two ways. First, we compared the distribution of recombination events on either side of each *P*[*w* ^+^] insert using the 67 complete sublines. If X-linked and autosomal introgressions are similar in size, we expect them to possess similar fractions of sublines with one or more recombination events within the ∼2-Mb window around the insert (1 Mb to the left and 1 Mb to the right). Consistent with this expectation, we find that X-linked and autosomal introgressions have a similar distribution of recombination events (*χ* ^2^ = 4.32, df = 2, *p =* 0.12). Second, we compared estimated introgression sizes between X-linked and autosomal introgressions using unpaired *t*-tests with null distributions generated from 1,000 randomizations of the data. We find no significant size difference between X-linked (mean ± one standard error = 1.33 ± 0.10 Mb; median = 1.46 Mb) and autosomal (1.49 ± 0.06 Mb; median = 1.47 Mb) introgressions using the 67 complete sublines (*p =* 0.15) or using all 108 sublines (*p =* 0.12); similar results hold when using nonparametric Mann-Whitney tests (*p*_complete_ = 0.33; *p*_total_ = 0.36).

Thus, the finding that X-linked introgressions cause hybrid male sterility more often than autosomal ones cannot be explained by a systematic difference in introgression size between the X chromosome and the autosomes. The probability that a D. mauritiana introgression will cause hybrid male sterility in D. sechellia is greater when the introgression resides on the X chromosome. Our results thus provide a proximate explanation for the large X effect: there is a higher density of hybrid male steriles on the X chromosome.

**Figure 4:**
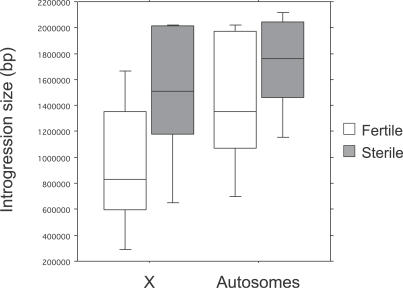
Distribution of Fertile and Sterile Introgression Sizes Boxes show the interquartile range; vertical lines are drawn out to the extreme values; horizontal lines within boxes show the median.

Interestingly, our data also show that hybrid male-sterile introgressions appear to be slightly larger than fertile introgressions. When we compare the distribution of recombination events among the complete sublines, we find no significant difference between fertile and sterile introgressions either within the X chromosome (*χ* ^2^ = 4.15, df = 2, *p =* 0.13) or within the autosomes (*χ* ^2^ = 1.41, df = 2, *p =* 0.49). However, in both comparisons we see a trend towards sterile introgressions being larger than fertile ones: 76% of sterile introgressions show one or more recombination events compared to 82% of fertile introgressions (Figure 4). Comparing mean size between fertile and sterile introgressions, we find no significant difference using the complete sublines (*p =* 0.11), but a marginally significant difference using all 108 sublines (*p =* 0.02). However, this marginally significant result does not affect our interpretation of the large X effect. Even if fertile and sterile introgressions differ slightly in size, introgression sizes on the X and the autosomes are the same.

### Estimating the Number of Hybrid Incompatibility Regions

Our data allow us to map the locations of 108 D. mauritiana introgressions, and thus obtain a rough estimate of the minimum number of hybrid inviable and hybrid male-sterile regions in the genome (Figure 2). We estimate that a minimum of four hybrid inviable regions separate D. sechellia and *D. mauritiana:* three on chromosome arm 3L and one on the X chromosome. We also estimate a minimum of eight hybrid male-sterile regions distributed roughly uniformly across the autosomal genome: three on chromosome arm 2L, zero on 2R, two on 3L, and three on 3R. Previous studies have shown that there are no hybrid inviables or hybrid male steriles on the small dot fourth chromosome between these species [75]. The average autosomal arm thus carries approximately two hybrid male-sterile regions. In contrast, a minimum of nine hybrid male-sterile regions are distributed over the length of the X chromosome—*more than four times the number on an average (and similarly sized) autosomal arm*. Thus, we find that at least one hybrid male sterility locus resides on each major chromosome, consistent with previous studies of postzygotic isolation in this species pair [76]. These numbers are, of course, minimum estimates for three reasons. First, each of our hybrid inviable or hybrid male-sterile introgressions might contain more than one hybrid incompatibility gene. Second, hybrid inviable introgressions may mask tightly linked hybrid male steriles. Third, we were unable to screen some regions of the genome and so may have missed some hybrid incompatible regions. However, because our coverage of the genome is fairly good, we do not expect that the qualitative difference—especially for hybrid male sterility—between the X and the autosomes would be much affected by higher resolution mapping.

### Evolutionary Basis of the Large X effect

Although dominance may contribute to the large X effect [10,16], our analysis distinguishes the *dominance* of hybrid male steriles from their relative *density* in two ways. First, the excess of hybrid male steriles on the X chromosome cannot be attributed to a methodological consequence of dominance [8,22] as we compare hemizygous X and homozygous autosomal effects. Second, we introgress more D. mauritiana material on the autosomes than on the X (97 versus 45 introgressions), thus exposing more potential recessive hybrid incompatibilities on the autosomes. Under the dominance theory (which assumes equal densities of hybrid incompatibilities on the X and on the autosomes [15,16]), we would expect to uncover more hybrid male steriles on the autosomes than on the X, in contrast to our findings.

Our introgression data demonstrate a higher density of hybrid male steriles on the X chromosome, but they do not explain *why* there are more on the X. We can exclude two explanations. First, there is not a higher concentration of male fertility-essential genes on the X chromosome. If anything, the opposite appears to be true in *Drosophila:* genes mutable to male sterility appear to be randomly distributed throughout the genome [77] and genes with male-biased expression are underrepresented on the X [78]. Second, the faster molecular evolution of X-linked loci does not appear to contribute to the large X effect. Charlesworth et al. [35] (see also [5]) showed theoretically that X-linked loci experience faster rates of substitution than autosomal loci when beneficial mutations are, on average, partially recessive. If true, we might expect the X chromosome to accumulate hybrid incompatibilities faster than equivalently sized autosomes. This theory predicts that X-linked loci will show greater sequence divergence between species than do autosomal loci. Population genetic tests for faster X evolution, however, show that the substitution rates of X-linked and autosomal loci in *Drosophila* are similar [36,37,79]. It is worth noting, however, that the effect of even a slightly elevated substitution rate on the X chromosome would be amplified, as the number of hybrid incompatibilities increases at least as fast as the square of divergence [16,80].

Three plausible explanations of the large X effect remain. First, recent discoveries of sex-ratio distortion in weakly fertile hybrid males [41,81,82] have renewed interest in the idea that genetic conflict might drive the evolution of X-linked hybrid male steriles [38,39]. Tao and Hartl [31] hypothesize that recurrent bouts of invasion by sex-chromosome meiotic drive loci can increase the density of hybrid incompatibilities on the sex chromosomes. They argue that because sex-ratio distorters affect gametogenesis, genes involved in conflict over sex ratio could have pleiotropic effects on fertility. As sex-ratio distorters usually reside on the X chromosome [39,83], this might give rise to a higher density of hybrid male steriles on the X versus the autosomes. One way to test for histories of sex-ratio conflict is to screen for sex-ratio distortion in species hybrids: distorters that are masked by suppressors in one species can be unmasked on the naïve genetic background of another species [41,82]. We therefore scored the sex ratio of 54 fertile introgression lines. We found only one subline, an autosomal introgression, that consistently produces a moderately male-biased sex ratio (Table S1). Further work, however, showed that this sex-ratio bias is not a hybrid phenomenon and does not involve sex-chromosome meiotic drive (data not shown). We thus conclude that there is no evidence for unmasked cryptic sex-ratio distortion among our fertile introgression lines. We cannot, of course, entirely exclude the possibility that past bouts of conflict have occurred in the D. mauritiana or D. sechellia lineages. Although no D. mauritiana autosomal introgressions released cryptic D. sechellia X-linked distorters, we were obviously unable to test most X-linked D. mauritiana introgressions for their ability to cause sex-ratio distortion in an otherwise D. sechellia background, as most of these introgressions were completely sterile.

A second possible explanation of the large X effect involves dosage compensation [5]. In particular, dosage compensation in the germline might be easily disrupted in hybrids. The X chromosome of *Drosophila* males is hyper-transcribed to equalize gene dose between males and females. In the soma, this process is under the control of the male-specific lethal (MSL) complex of proteins [84]. Divergence of the MSL machinery between species could thus cause a breakdown in dosage compensation in interspecific hybrids. Because this would affect nearly all X-linked loci, the X chromosome would have a disproportionately high density of hybrid incompatibilities. Although previous studies provide evidence that disruption of the MSL-mediated dosage compensation pathway does not cause inviability in *D. melanogaster–D. simulans* hybrids [85], recent work suggests that dosage compensation does occur in the germline by an MSL-independent mechanism [86]. A breakdown of dosage compensation specifically in the germline could, therefore, potentially produce a large X effect for hybrid male sterility.

Finally, X inactivation—the condensation of the X chromosome during early spermatogenesis—could be disrupted in hybrid males. It has been suggested that spermatogenesis may be an inherently sensitive developmental process, rendering hybrid males particularly prone to sterility [8,30]. Genetic and cytogenetic evidence within species supports this idea: spermatogenesis appears sensitive to genetic perturbations, particularly with respect to the X chromosome [87]. In *D. melanogaster,* for instance, translocations from the autosomes to the X almost always result in dominant male sterility. Sterility in these cases is thought to result from improper X inactivation in primary spermatocytes [87]. It seems possible, then, that foreign genetic material that is recognized as “non-X” by the X inactivation machinery could disrupt X inactivation, causing hybrid male sterility. If introgressions on the X from one species are not recognized as X-linked material by the inactivation machinery of the other species, X-linked introgressions could result in hybrid male sterility, giving rise to a large X effect. The present data do not allow us to distinguish among the three potential evolutionary causes of the large X effect presented here. Resolution of the ultimate cause must, therefore, await further molecular studies.

## Materials and Methods

### Drosophila stocks.

Construction of the *D. mauritiana–D. sechellia* introgression lines was performed using *D. sechellia w* (kindly provided by J. A. Coyne, University of Chicago) and the *D. mauritiana P*-element insertion stocks described in True et al. [21,74]. Each of these stocks contains a single *P*[*lac-w* ^+^] insertion in a *D. mauritiana w* background. The *P*[*w* ^+^] insert acts as a semi-dominant visible marker; flies heterozygous for the insert on a *w* background show orange eye color, whereas flies homozygous for the insert show red eye color. The inserts are randomly distributed over all four chromosomes at 87 known cytological positions [21].

### Introgression procedure.

Figure 1 shows our introgression procedure, which closely followed that of True et al. [21]. We began with the 84 (of the original 103) *P*[*w* ^+^] inserts that are still in existence. Briefly, fertile F_1_ hybrid females were generated by crossing *D. sechellia w* females to D. mauritiana males homozygous for the *P*[*w* ^+^] markers. We established 2–4 independent replicate sublines for each *P*[*w* ^+^] insert by crossing F_1_ hybrid females to *D. sechellia w* males. Each subline was then backcrossed independently for 15 generations by crossing hybrid females heterozygous for the *P*[*w* ^+^] insert with *D. sechellia w* males. This crossing scheme produced hybrid introgression lines that have a mostly D. sechellia genetic background, but that carry a small chromosome region from D. mauritiana marked by the *P*[*w* ^+^] insert.

To make introgressions homozygous, five heterozygous (hemizygous) male progeny were selected from the G15 backcross and mated individually to ten *D. sechellia w* virgin females (cross H1; Figure 1B and 1C); five white-eyed male siblings were also mated individually to ten *D. sechellia w* virgin females as controls. (These white-eyed males share the same genetic background as their *P*[*w* ^+^] siblings, including any unmarked D. mauritiana material that is not linked to the *P*[*w* ^+^] insert. So long as there is no unmarked D. mauritiana material, these white-eyed males should be fertile. Indeed, these control males were always fertile.) If at least one H1 hybrid male produced offspring, progeny from a single fertile male were selected for cross H2.

For autosomal introgressions (Figure 1B), if cross H2 proved fertile, homozygous progeny were scored for viability and fertility (crosses H3). If homozygous viable and fertile H2 progeny of both sexes were produced, they were crossed to establish a homozygous hybrid introgression line.

For X-linked introgressions (Figure 1C), heterozygous H2 females were crossed with *D. sechellia w* males to produce heterozygous females and hemizygous males. These flies were used in cross H3. If the resulting homozygous female progeny were viable and fertile, they were crossed with their hemizygous brothers to establish a homozygous hybrid introgression line (cross H4). For most X-linked introgressions, however, all five H1 hybrid males failed to produce offspring. Therefore, hybrid male fertility was also tested in mass matings by crossing ten hemizygous hybrid males with ten *D. sechellia w* virgin females (H5).

### Testing viability and fertility.

Viability and fertility were scored after autosomal (Figure 1B) and X-linked (Figure 1C) introgressions were made homozygous. Each introgression subline was classified into one of four categories: lethal, female-sterile, male-sterile, or fertile. Hybrid lethality was scored as the absence of red-eyed progeny of both sexes from cross H2 for autosomal introgressions, or the absence of red-eyed males from backcross G15 for X-linked introgressions (Figure 1). Homozygous hybrid male and hybrid female fertility were measured as the ability to produce offspring in mass matings with *D. sechellia w* flies. Crosses that did not produce progeny were scored as sterile. Because of the large number of crosses performed in a short period of time, we were unable to simultaneously score sperm motility, as is commonly done.

For some inserts, it was impossible to distinguish homozygous individuals from heterozygous individuals. For these cases, male and female progeny from cross H2 (autosomes) and cross H3 (X) were mated individually with *D. sechellia w* females and males, respectively, to test their viability and fertility. Homozygous and heterozygous individuals could then be distinguished by progeny testing, as segregation of the *P*[*w* ^+^] marker will only occur among the progeny of heterozygotes.

### Genotyping introgression breakpoints.

The *D. mauritiana P*[*w* ^+^] inserts were localized originally to the resolution of cytological bands [21,74]. Recently, however, flanking regions from 95 inserts have been sequenced and were kindly provided by Y. Tao (Emory University) and L. Araripe (Harvard University). These data provide precise genomic locations for each *P*[*w* ^+^] insert. At the time of our analyses, the genome sequence for D. sechellia was not available. Because both D. mauritiana and D. sechellia are closely related to (and homosequential with) D. simulans (1%–2% sequence divergence), we used the D. simulans genome assembly to obtain genomic coordinates for the sequenced inserts.

We used species-specific microsatellite repeat length differences as markers to roughly determine introgression sizes. We identified potential markers approximately 1 Mb, 500 kb, and 100 kb to the left and to the right of each *P*[*w* ^+^] insert using Tandem Repeats Finder [88] (Figure S1). We then designed primers flanking microsatellites from D. simulans genomic sequence. We used a standard protocol to amplify markers, and PCR fragments were separated on 8% polyacrylamide gels to identify length differences. Although we were unable to find suitable markers for every region, in several cases the genotype at those locations could be inferred from the genotypes at adjacent markers. When scoring markers, we assumed that unobserved double recombination events are sufficiently rare as not to occur between adjacent markers (i.e., no double crossovers within ∼500-kb windows). When we could not score or reliably infer genotype, species identity was treated as unknown. We were able to score a total of 485 microsatellite markers from 55 *P*[*w* ^+^] insert regions.

### Introgression size estimation.

Because we know the genomic coordinates of the *P*[*w* ^+^] inserts and the microsatellite markers, we can obtain good estimates of introgression size for each subline. We calculated a minimum and a maximum size of D. mauritiana material within a 2-Mb region around each insert (Figure S1). With respect to the insert's location, minimum introgression size was calculated as the distance between the farthest markers to the left and the right sides of the insert that showed a D. mauritiana allele. Maximum introgression size was calculated as the distance between the nearest markers to the left and the right sides of the insert that showed a D. sechellia allele.

Twenty-two sublines showed the D. mauritiana allele at all six markers. For these cases, minimum introgression size was calculated as the distance between the left- and right-most distal markers (L3 and R3, respectively), and the maximum introgression size was calculated by adding two base pairs to this distance. Likewise, five sublines showed the D. sechellia allele at all six markers. In this case, maximum size was calculated as the distance between the left- and right-most proximal markers (L1 and R1, respectively), and minimum size was calculated as two base pairs.

Because our introgression size estimates have a maximum size limit of roughly 2 Mb, our genotype data might underestimate true introgression size. The distribution of recombination events within this 2-Mb window, however, shows that 81% (54/67) of the complete sublines experienced at least one recombination event and 48% experienced two recombination events. Thus, our data appear to capture a reasonably accurate sample of introgression size. We present the results from our statistical tests using the maximum size estimates for simplicity. Our conclusions do not depend on the scoring procedure because the results using minimum estimates of introgression size remain similar.

The distributions of introgression sizes are non-normal. We therefore tested for differences in means (e.g., X versus autosomes or fertile versus sterile) using unpaired *t*-tests with null distributions generated by 1,000 randomizations of the data. Means are reported ± one standard error.

## Supporting Information

Figure S1 Estimation of Introgression Breakpoints The horizontal bar shows a hypothetical introgression: D. sechellia material is shown in white; D. mauritiana material is shown in gray; *P*[*w* ^+^] insert is shown with an inverted black triangle. Uncertainty in introgression breakpoint location is indicated with stripes. Markers are shown above the introgression, and their approximate distances from the *P*[*w* ^+^] insert are shown below. Minimum introgression size is calculated as the distance between markers L2 and R1, and maximum distance is calculated as the distance between markers L3 and R2. Gel image shows the appearance of this introgression when genotyped: h, allele carried by the hybrid introgression line; m, D. mauritiana allele; s, D. sechellia allele.
(166 KB PDF)

Table S1 Postzygotic Reproductive Isolation in Drosophila mauritiana–D. sechellia Introgression Lines Sex ratios for viable, fertile introgressions are reported as proportion male. FF, female fertile; LETHAL, inviable; LINE, homozygous viable and fertile introgression line established; MF, male fertile; MS, male sterile; ND, not determined; WFF, weakly female fertile; WMF, weakly male fertile.
(36 KB XLS)

## Abbreviations

MSL: male-specific lethal
