# Supplementary material for: High-Resolution Genome-Wide Dissection of the Two Rules of Speciation in Drosophila
Source: PLoS Biol. 2007 Sep 11;5(9):e243. doi: 10.1371/journal.pbio.0050243 (PMC1971125; doi:10.1371/journal.pbio.0050243)
Supplement: Supplementary file 1 [file pbio.0050243.sg001.pdf]

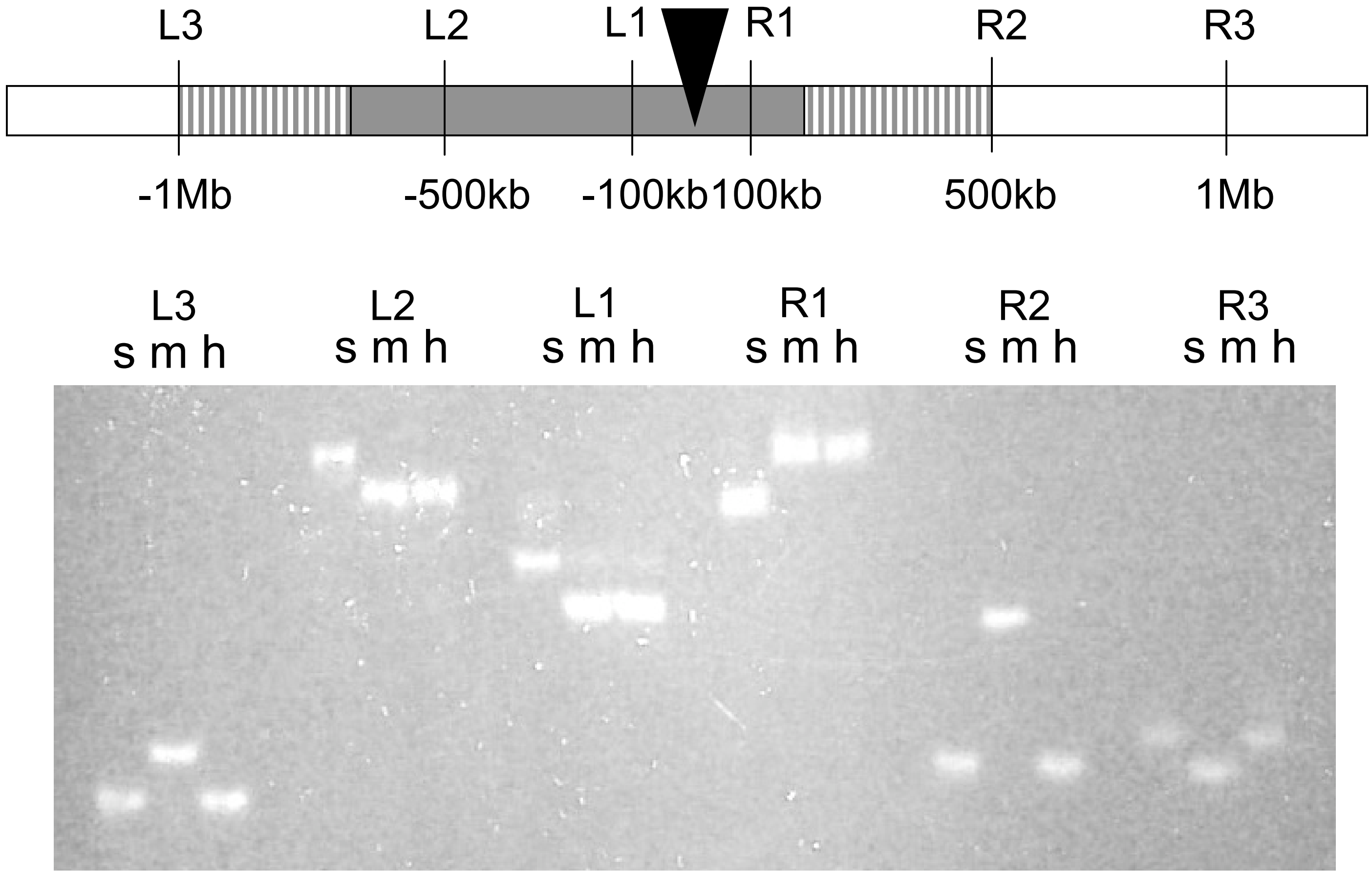
L3
L2
L1
R1
R2
R3
-1Mb
-500kb
-100kb
100kb
500kb
1Mb
L3
s m h
L2
s m h
L1
s m h
R1
s m h
R2
s m h
R3
s m h
